# Comparison of Two Types of Guidewires for Malignant Hilar Biliary Obstruction by Endoscopic Retrograde Cholangiopancreatography: A Randomized Controlled Trial

**DOI:** 10.3390/jcm12103590

**Published:** 2023-05-22

**Authors:** Sung Yong Han, Jung Wan Choe, Dong Uk Kim, Jong Jin Hyun, Joung-Ho Han, Hoonsub So, Sung Jo Bang, Dong Hee Koh, Seok Jeong

**Affiliations:** 1 Department of Internal Medicine, Pusan National University School of Medicine, Biomedical Research Institute, Pusan National University Hospital, Busan 49421, Republic of Korea; mirsaint@hanmail.net; 2 Department of Internal Medicine, Korea University Ansan Hospital, Ansan 15355, Republic of Korea; jwchoe@korea.ac.kr (J.W.C.); sean4h@korea.ac.kr (J.J.H.); 3 Department of Internal Medicine, Chungbuk National University College of Medicine, Chungbuk National University Hospital, Cheongju 28644, Republic of Korea; joungho@chungbuk.ac.kr; 4 Department of Internal Medicine, Ulsan University Hospital, University of Ulsan College of Medicine, Ulsan 05505, Republic of Korea; hoon3112@gmail.com (H.S.); sjbang@uuh.ulsan.kr (S.J.B.); 5 Department of Internal Medicine, Hallym University Dongtan Sacred Heart Hospital, Hwasung 18450, Republic of Korea; donghee73@hanmail.net; 6 Department of Internal Medicine, Inha University Hospital, Inha University School of Medicine, Incheon 22212, Republic of Korea

**Keywords:** malignant hilar biliary obstruction, guidewire, endoscopic retrograde cholangiopancreatography

## Abstract

**Background:** There is insufficient information regarding the optimal guidewire for managing malignant hilar biliary obstruction (MHBO). Therefore, a newly designed 0.025-inch guidewire was compared with the conventional 0.035-inch guidewire for selective cannulation of both intrahepatic ducts (IHDs) in patients with MHBO. **Methods:** Patients were randomly enrolled into the curved type newly designed 0.025-inch guidewire group (0.025 group) or the curved type conventional 0.035-inch guidewire group (0.035 group). The primary outcome was the selective cannulation rate of IHD. If the assigned guidewire failed to pass the stricture within 5 min, the crossover guidewire was selected. If the crossover guidewire failed to cross the stricture within the next 5 min, it was judged as a failed selective cannulation of both IHDs. **Results:** A total of 90 patients were enrolled (0.025 group, *n* = 47; 0.035 group, *n* = 43). There was no significant difference in baseline characteristics between the groups regarding sex, age, BMI, obstruction level, and clinical presentation. Four patients (8.5%) in the 0.025 group the cannulation of the IHD failed and the conventional 0.035-inch guidewire was substituted in a second attempt; the 0.035-inch guidewire failed to cross the stricture in all four patients. In the 0.035 group, eleven patients (25.6%) failed to achieve selective cannulation of IHD, and the 0.025-inch guidewire was substituted; the newly designed 0.025-inch guidewire crossed the stricture in ten of these (10/11, 90.9%). The selective cannulation rate of IHD was significantly higher in the 0.025 group (95.1% vs. 85.5%, *p* = 0.043). **Conclusions:** The 0.025 group exhibited a higher success rate for selective cannulation of both IHDs in MHBO than did the 0.035 group.

## 1. Introduction

Endoscopic retrograde cholangiopancreatography (ERCP) is a standard technique for diagnosing and treating pancreatobiliary disease. A guidewire is key for inserting or maintaining various devices in the bile duct or pancreatic duct during a treatment procedure. Especially in malignant hilar biliary obstruction (MHBO), bilateral drainage provides superior drainage effectiveness and survival [1,2]. The Asia-Pacific Working Group and ESGE guidelines recommend draining over 50% of the liver volume [3,4]. Bilateral drainage is commonly required to achieve this result. However, it could be difficult to cannulate the contralateral intrahepatic duct (IHD) in some patients after cannulating one IHD, despite multiple manipulations [5]. Bilateral drainage depends on the success of the selective cannulation of contralateral IHD after selecting one IHD, and optimal guidewire selection is the mainstay of success.

**Figure 1:**
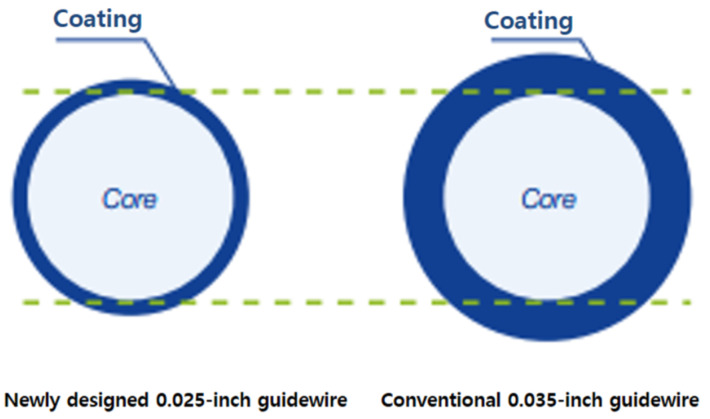
Two types of guidewire used in the study.

There are various guidewires with differing characteristics, such as tip shape, flexibility, stiffness, hydrophilic coating, etc. Two main guidewires are used worldwide: the 0.025-inch and 0.035-inch guidewires. The hydrophilic tip portion is longer in the newly designed 0.025-inch guidewire than the conventional 0.035-inch guidewire [6]. Hydrophilic coating lubricates the wire, and a special fluorine coating reduces friction, making device manipulation effortless and allowing for easy navigation of the ductal structure. The core wire is associated with stiffness. The newly designed 0.025 guidewire has a similar core wire, including the same stiffness as a conventional 0.035-inch guidewire (Figure 1). Therefore, theoretically, the 0.025-inch guidewire may be better for crossing the MHBO. However, there is limited research regarding the optimal guidewire for selective cannulation of both IHDs in patients with MHBO. Therefore, we aimed to compare the 0.025-inch and 0.035-inch guidewires to evaluate the success rate of selective cannulation of both IHDs in patients with MHBO.

## 2. Methods

### 2.1. Study Design and Sample Size Calculation

We performed a randomized controlled trial to compare two types of curved guidewires in MHBO. The patients were simultaneously enrolled at six referral hospitals. Randomization was performed by opening the randomization envelope after the patient completed the informed consent. The trial started in March 2020, and the final patient enrollment was closed in February 2022.

The enrollment criteria were (1) patients with MHBO requiring ERCP for biliary drainage or diagnosis via biopsy, (2) patients needing a guidewire during the ERCP, and (3) patients who had never had a guidewire or catheter passed through the MHBO before the procedure. The exclusion criteria were (1) surgically altered anatomy (such as Billroth I or II, Roux-en-Y anastomosis, etc.), and (2) patients with distal bile duct obstruction.

A previous study compared two types of guidewires in biliary stricture (0.035 inch and hydrophilic wire covered with polyurethane and hydrophilic coating) [7]. Therefore, we used the parameters in that article (bile duct stricture passage rate of 94%) and planned a non-inferiority test (non-inferiority margin of 10%), using calculators in powerandsamplesize.com (alpha to 0.05 and beta to 0.20). Each group was calculated at 70 patients. A total of 140 patients were needed, and we considered failed cannulation as failure to reach the bile duct. Moreover, we used 82.5% as a cannulation success rate because the previous article indicated an 80–85% success rate [8]. Thus, a total of 170 patients were required for enrollment. An interim analysis was performed when half of the patients were enrolled. We obtained results showing that one group exhibited significant superiority. Hence, we stopped further enrollment. This study was conducted in accordance with the ethical guidelines of the Declaration of Helsinki (revised in 2013), and the study protocol was approved by the Pusan National University Hospital Institutional Review Board (no. 1810-028-071, approved on 26 October 2018). The Clinical Research Information Service (CRIS) also approved this study (no. KCT0003510).

### 2.2. Definitions

The primary outcome was the selective cannulation rate of the IHD before and after crossover. The important primary outcome is the stricture passage rate before crossover. Additionally, another primary outcome is the stricture passage rate after crossover because it was important to determine whether the failed cases did not pass due to severe stricture, or due to the characteristics of the guidewire when evaluating its performance. Patients with a bilateral drain due to Bismuth-Corlette classification type (B-) II/III/IV were defined as having two strictures, and patients with a single drain were defined as having one stricture. Secondary outcomes were the complete drainage rate and the adverse event rate. The complete drainage rate was defined as bilateral drainage with one (Bismuth I) or two stents (Bismuth II, III, IV). The lexicon for endoscopic adverse events was used to classify adverse events and their severity [9].

### 2.3. Procedure

All procedures were performed using Olympus duodenoscopes (Olympus scopes; TJF 240, TJF 260, JF 240, JF 260; Olympus, Tokyo, Japan). The choice of the catheter was determined according to the preference of the endoscopists and included balloon catheters, sphincterotomes, cannulas, etc. Patients were randomly enrolled into the newly designed 0.025-inch guidewire (VisiGlide-2, curved type, 450 cm, Olympus Co., Tokyo, Japan) group (0.025 group) or the conventional 0.035-inch guidewire (Jagwire, curved type, 450 cm, Boston Scientific, Marlborough, MA, USA; Tracer metro, curved type, 450 cm, Cook Medical, Bloomington, IN, USA) group (0.035 group). After successful common bile duct (CBD) selective cannulation, cholangiography was obtained. For bilateral drainage, if the extent was Bismuth I, one plastic stent was deployed after passing the stricture because in patients diagnosed as Bismuth I could achieve bilateral drainage with one plastic stent. If the extent was B II, III, or IV, the bilateral selection of IHD (left and right anterior or left and right posterior) was attempted. The stricture passage was attempted with each selected guidewire. After selecting both IHDs, plastic stents were deployed at each selected IHD, and we only inserted a plastic, not a metal, stent. If the randomly selected guidewire failed to pass the targeted stricture within 5 min, the guidewire was changed to another type (0.025 to 0.035 and vice versa). If the changed guidewire failed to cross the MHBO within the next 5 min, it was judged to be a procedural failure. The endoscopist could decide on an additional procedure, such as the use of a swing tip catheter, percutaneous transhepatic drainage, etc. All the procedures were performed by experts (a total of seven endoscopists in six referral hospitals) who had performed at least 3000 ERCP procedures; fellows were not involved.

### 2.4. Statistical Analysis

Statistical analysis was performed using the SPSS software (version 21.0, IBM Corp., Armonk, NY, USA). Categorical data were expressed as frequency and percentage, and between-group differences were evaluated using the Chi-square test. Continuous data were expressed as mean ± standard deviation (SD), with between-group differences evaluated using an independent Student’s *t*-test. Statistical significance was set at *p* < 0.05.

## 3. Results

### 3.1. Baseline Characteristics

A total of 90 patients were enrolled; 47 patients were in the 0.025 group (Bismuth types I, *n* = 13; II, *n* = 9; IIIA, *n* = 10; IIIB, *n* = 4; IV, *n* = 11), and 43 were in the 0.035 group (Bismuth types I, *n* = 7; II, *n* = 9; IIIA, *n* = 16; IIIB, *n* = 2; IV, *n* = 9). Age, sex, body mass index, diagnosis, obstruction level, clinical presentation, and pre-procedure laboratory findings were not statistically different between the groups. Factors related to ERCP, including peri-ampullary diverticulum, pancreatic duct stenting, post-procedure laboratory finding, cannulation time, cannulation method, and adverse events, were also not significantly different. The total procedure time was significantly longer in the 0.035 group (1243.7 vs. 1539.4 s, *p* = 0.020) (Table 1).

### 3.2. Study Flow Chart

**Figure 2:**
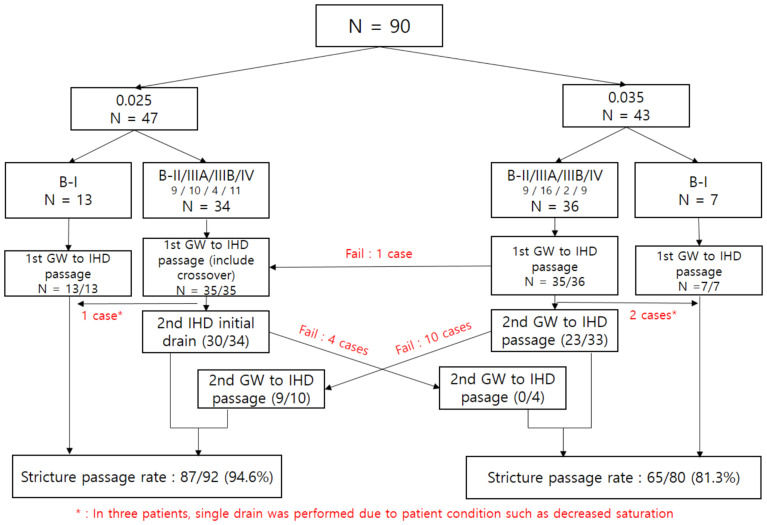
Flow chart of study; IHD, intrahepatic duct; B-, Bismuth; GW, guidewire.

In the 0.025 group, single drainage for B-type I was successful for 13 patients. The first guidewire was used to cannulate the IHD in 34 patients with B-II/III/IV, and all were successful. Subsequently, in the 34 patients, a second trial was performed on the opposite side of the first guidewire that cannulated the IHD. In 30 patients, selective cannulation for the second guidewire to cannulate the IHD was successful, but cannulation was impossible within the time limit of 5 min in four patients. Selective cannulation for the second guidewire to the IHD was not achieved, even with a crossover 0.035-inch guidewire in the four failed patients (Figure 2).

In the 0.035 group, single drainage was performed successfully in all seven patients with B-type I. The other cannulation of the first guidewire to the IHD was successful in the 36 patients with B-II/III/IV, except for 1 case. The failed case was successful with the crossover trial using the 0.025-inch guidewire. In the two patients requiring a selective second guidewire cannulation to the IHD, based on B-II/III/IV findings, the procedure was terminated with one drainage, considering the patient’s condition. Of the 34 patients who continued the procedure for the second guidewire cannulation to the IHD, 10 failed the procedure, and the crossover was performed with the 0.025-inch guidewire. Among the 10 patients, 9 obtained success with the crossover 0.025-inch guidewire. However, in one patient, the attempt failed, even after using the 0.025-inch guidewire (Figure 2).

### 3.3. Primary Outcome

The success rate of the 0.025-inch guidewire for MHBO’s first guidewire selective cannulation to the IHD was 100% (48/48), including one patient who failed with the 0.035-inch guidewire for first guidewire cannulation to the IHD and was rescued with the crossover of the 0.025-inch guidewire. In the selective second guidewire cannulation to the IHD with the 0.025-inch guidewire, 39 out of 44 patients (34 in the 0.025 group and 10 patients in the 0.035 group in whom the crossover was performed) showed successful passage.

The final successful penetration rate of the malignant hilar biliary stricture using the 0.025 guidewire was 94.6% (87/92). The success rates of the first and second guidewire selective cannulation to IHD in MHBO with the 0.035 guidewire were 97.7% (42/43) and 73.0% (27/37). The final success rate in the hilar biliary stricture with the 0.035-inch guidewire was 81.3% (65/80). Guidewire crossover occurred more frequently in the 0.035 group (8.5% vs. 25.6%, *p* = 0.039). Before crossover, the success rate was 95.1% in the 0.025 group and 85.5% in the 0.035 group (*p* = 0.043). After crossover, the success rate was 94.6% in the 0.025 group and 81.3% in the 0.035 group (*p* = 0.006). These rates were significantly statistically different.

### 3.4. Secondary Outcomes

**Figure 3:**
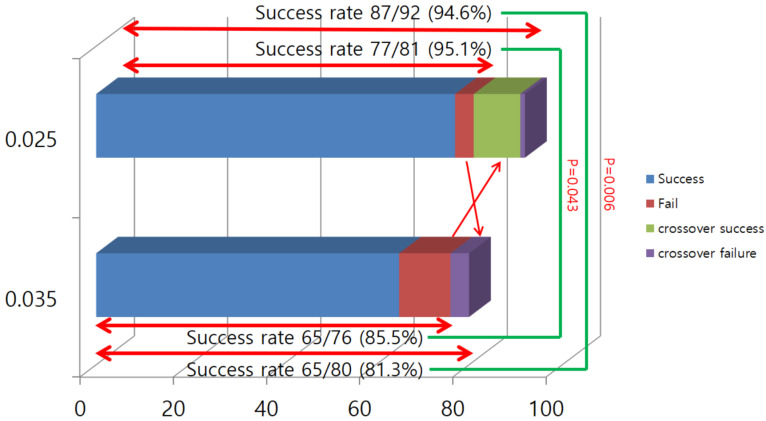
Stricture passage rate in the 0.025 and 0.035 groups.

The complete drainage rate was different between both groups (91.5% vs. 74.4%, *p* = 0.030) (Figure 3, Table 2). Table 3 shows the clinical information for patients with crossover. A total of 15 patients experienced crossover, 11 in the 0.035 group and 4 in the 0.025 group. Almost all patients were diagnosed with cholangiocarcinoma and B-III or IV. There was no difference in the success rate according to the types of IHD, and crossover was performed at the insertion of the second guidewire, except in one patient. Insertion of the guidewire after crossover failed in five patients, four in the 0.025 group and one in the 0.035 group. There was no statistical difference in adverse events, including the delayed adverse events, between the groups.

## 4. Discussion

The newly designed 0.025-inch guidewire exhibits a higher stricture passage rate (95.1% vs. 85.5%, *p* = 0.043) and a higher complete drainage rate (91.5% vs. 74.4%, *p* = 0.030) for Klatskin tumors than the conventional 0.035-inch guidewire. After adding the crossover cases, the stricture passage rate was more statistically significantly different (94.6% vs. 81.3%, *p* = 0.006). Four crossover cases (8.5%) were in the 0.025 group, all of which also failed with the 0.035-inch guidewire. There were 11 crossover cases (25.6%) in the 0.035 group. In all but one case, the stricture passage was successful in the retry using the 0.025-inch guidewire. Crossover occurred mainly in the second IHD assessment (14/15, 93.3%). The total procedure time was longer in the 0.035 group (1243.7 ± 531.7 vs. 1539.4 ± 647.0 s, *p* = 0.020). The adverse event rates were not statistically significantly different between the groups.

An endoscopist considers two important factors when using a guidewire: pushability and torquability. Generally, the thinner the guidewire, the better it will pass through the narrow stricture space. However, if the diameter of the guidewire is too thin, the stiffness is low, and it becomes difficult to transmit the force to the end through a 450-cm-long guidewire. If the stiffness is lowered, the force transmitting power is lowered, which can decrease pushability and torquability. To overcome this hurdle, it was necessary to develop a new guidewire to maintain the stiffness of the existing 0.035 guidewire and make the diameter as thin as possible to pass through the narrow stricture. Accordingly, the newly designed 0.025-inch guidewire used in this study has a core diameter similar to that of the conventional 0.035-inch guidewire [6,10].

Another factor that may affect passage through the biliary stricture is the hydrophilicity of the tip of the guidewire. The 0.025-inch and 0.035-inch guidewires used in this study have hydrophilic tips of different lengths. The 0.025-inch guidewire has a 7 cm hydrophilic tip, and the 0.035-inch guidewire has a 5 cm hydrophilic tip at the distal end. The longer length of the hydrophilic coated portion at the distal end will be more helpful in sliding the guidewire through the biliary stricture, which is considered one of the reasons for the difference in the rate of successful passage in this study.

In our study, guidewires were inserted into the IHD, and most cases required two guidewires to penetrate through the stricture. All crossover cases occurred at the second guidewire insertion, except for one. In MHBO, the IHD is angled and difficult to pass through. Therefore, torque is very important for MHBO passage. Although it was impossible to measure objectively, all the endoscopists in our study declared that the torque of the second insertion of the 0.035-inch guidewire was difficult. If two 0.035-inch guidewires were included in the stricture compared to the 0.025-inch guidewire, the friction would increase, and this may reduce the torque capability of the angled tip. Even when the 0.035-inch and 0.025-inch guidewires were inserted simultaneously after crossover, all the cases ultimately failed when the second guidewire was the 0.035 inch type; when the second guidewire was the 0.025-inch type, 9 of 10 cases were successful. Therefore, the 0.025-inch guidewire is considered better for the performance of the second guidewire insertion.

Post-ERCP pancreatitis, bleeding, and perforation are the most common adverse events in ERCP; these mainly occur in biliary cannulation [11]. Thus, compared with the passage rate of MHBO, the adverse events rate was not as important in biliary cannulation. Our results showed similar adverse event rates between both groups, and bile duct injuries, such as bile duct perforation by a guidewire, did not occur in any patient. All patients underwent successful biliary cannulation, even though each endoscopist may choose different devices for cannulation, and this may impact the success rate. There was no restriction on the cannulation method and time, which might explain the 100% successful cannulation rate. All the procedures were performed by experts who had performed at least 3000 ERCP procedures; fellows were not involved. Guidewire manipulation was performed by an expert nurse or endoscopist. Difficult cannulation occurs in a surgically altered anatomy or near the ampullary invasion of malignancy [12]. However, such cases were excluded from our study.

This study did not investigate the adequacy of the guidewire according to the type during cannulation. The strength of our study is that it confirms the passage success rate in the stricture of the hilar area. Although previous studies have been conducted to evaluate the difference in the procedure through the characteristics of these types of guidewires, most of them are only studied for biliary cannulation [8,10,13,14,15]. Most studies showed similar cannulation and adverse event rates, so we assumed there was no significant difference in the results according to the differences between these guidewires in biliary cannulation. However, studies regarding the success rate of passage through a stricture in patients with Klatskin tumors have not yet been reported, and this is a very challenging field for endoscopists. In addition, this study is an objective multicenter study with a relatively large number of cases.

Our study has some limitations. First, the results showed a significant difference in the second IHD access. However, the proportion of patients in whom a single drainage was performed was high. Statistically, more meaningful results could have been obtained if only patients requiring bilateral drainage were included. However, it was possible to show a statistically significant difference, even with the current number of patients. Second, various types of catheters were allowed, which may have affected the results. However, it is judged that the effect was minimized because there was no difference in action, such as changing the catheter’s angle by manipulating the nose of the sphincterotome, and the ratio of catheter was not statistically different between two groups. Third, we planned for 170 patients to be enrolled; however, only 90 patients were actually enrolled. Interim analysis was performed, showing significant differences between the two groups; however, these results were not sufficient for discontinuing the study. If we enrolled 170 patients as our sample size calculation, it is possible that there may have been a change in the results. However, 140 patients were needed to reveal the efficacy of guidewire use in a hilar malignant stricture, we enrolled a second third of planned number.Moreover, all of our results for stricture passage and complete drainage rates were significantly different between the two groups.

In conclusion, the newly designed 0.025-inch guidewire has a higher passage success rate in MHBO than the conventional 0.035-inch guidewire. Passage with the 0.035-inch guidewire is more difficult in the second guidewire cannulation to MHBO than with the 0.025-inch guidewire. According to our results, the newly designed 0.025-inch guidewire is more suitable for complete drainage in MHBO. Further studies are needed to prove the results.

## Figures and Tables

**Table 1:** Baseline characteristics and result of ERCP.

	0.025 Group, *n* = 47	0.035 Group, *n* = 43	*p*-Value
Sex, male (%)	24 (51.1)	23 (53.5)	0.821
Age	71.6 ± 12.0	75.2 ± 8.7	0.098
BMI	22.5 ± 4.7	23.6 ± 3.1	0.171
Diagnosis (*n*) CCA/GB ca/others	35/12/0	38/4/1	0.203
Obstruction level B-I/-II/-IIIA/-IIIB/IV	13/9/10/4/11	7/9/16/2/9	0.689
Clinical presentation Pain/jaundice/fever/others	7/32/3/5	5/29/1/8	0.350
Peri-ampulary diverticulum	5 (10.6)	4 (9.3)	0.681
Pancreatic duct stenting	15 (32.6)	18 (41.9)	0.372
Protease inhibitor	40 (87.0)	37 (86.0)	0.901
Antibiotics	44 (93.6)	39 (90.7)	0.610
**Pre-laboratory findings**			
WBC	8295.3 ± 6043.9	9031.1 ± 10,097.6	0.673
Total Bilirubin	12.6 ± 15.7	10.3 ± 7.1	0.368
C-related Protein	3.71 ± 4.49	2.72 ± 2.98	0.228
**Post 4 h lab**			
WBC	10,395.5 ± 14,417.0	7988.1 ± 3287.1	0.288
Amylase	213.0 ± 343.5	188.9 ± 455.6	0.778
Lipase	412.8 ± 982.6	339.4 ± 921.6	0.604
**Post 1 day lab**			
WBC	8572.81 ± 3809.5	9238.3 ± 3368.1	0.390
Total Bilirubin	7.20 ± 6.61	7.88 ± 6.75	0.630
Amylase	370.9 ± 709.1	297.5 ± 723.5	0.638
Lipase	428.4 ± 1031.4	422.7 ± 783.8	0.634
**ERCP Procedure**			
Cannulation time	245.9 ± 263.3	322.6 ± 281.9	0.185
Total procedure time	1243.7 ± 531.7	1539.4 ± 647.0	0.020 *
Catheter (sphincterotomy/cannula)	36 (76.6)/11 (23.4)	31 (72.1)/12 (27.9)	0.629
Primary cannulation method Guidewire/NKF	42 (89.4)/5 (10.6)	39 (90.7)/4 (9.3)	0.835
Rescue method Septotomy/duobleGW/NKF	15 (31.9) 5/8/2	19 (44.2) 6/10/3	0.859
**Adverse events**			
Post ERCP pancreatitis	3 (6.4)	5 (11.6)	0.388
Asymptomatic Hyperamylasemia	10 (21.3)	9 (20.9)	0.968
Bleeding	0 (0)	3 (7.0)	0.067

BMI, body mass index; CCA, cholangiocarcinoma; GB ca., gallbladder cancer; B-, Bismuth; NKF, needle knife fistulotomy; GW, guidewire; * *p*-value < 0.05.

**Table 2:** Result of guidewire passage for MHBO.

	0.025 Group, *n* = 47	0.035 Group, *n* = 43	*p*-Value
1st GW cannulate to IHD (S)	39.2 ± 47.9	41.7 ± 58.8	0.821
2nd GW cannulate to IHD (S)	138.2 ± 182.5	171.6 ± 189.7	0.452
2nd GW cannulate to IHD, without failed cases (S)	104.8 ± 103.8	132.6 ± 122.1	0.306
1st GW cannulate to IHD Rt. Ant/Rt. Post/Lt. IHD	23 (48.9)/5 (10.6)/19 (40.4)	21 (48.8)/4 (9.3)/18 (41.9)	0.940
2nd GW cannulate to IHD Rt. Ant/Rt. Post/Lt. IHD	13 (27.6)/2 (4.3)/14 (29.8)	13 (30.2)/4 (9.3)/16 (37.2)	0.404
Guidewire crossover (1st/2nd)	0(0)/4 (8.5)	1 (2.3)/10 (23.3)	0.039 *
Guidewire stricture passage	77/81 (95.1)	65/76 (85.5)	0.043 *
Guidewire stricture passage with crossover cases	87/92 (94.6)	65/80 (81.3)	0.006 *
Complete drainage rate	43/47 (91.5)	32/43 (74.4)	0.030 *

IHD, intrahepatic duct; GW, guidewire; Rt. Ant, right anterior; Rt. Post., right posterior; Lt., left; * *p*-value < 0.05.

**Table 3:** Clinical information of patients with crossover.

No	Age	Sex	1st Guidewire	Diagnosis	Obst Level	Crossover	1st IHD	Time (S)	2nd IHD	Time (S)
1	81	Female	0.035	CCA	B-IIIA	2nd GW/0.025	Lt. IHD	5	Rt. Ant	300 + 60
2	80	Male	0.035	CCA	B-II	2nd GW/0.025	Lt. IHD	30	Rt. Ant	300 + 185
3	78	Female	0.035	CCA	B-IV	2nd GW/0.025	Lt. IHD	30	Rt. Ant	300 + 180
4	60	Male	0.035	CCA	B-IV	2nd GW/0.025	Rt. Ant	5	Lt. IHD	300 + 240
5	71	Female	0.035	CCA	B-II	2nd GW/0.025	Rt. Ant	150	Lt. IHD	300 + 10
6	71	Female	0.035	CCA	B-IV	2nd GW/0.025	Rt. Ant	10	Lt. IHD	300 + 240
7	72	Male	0.035	CCA	B-IIIA	2nd GW/0.025	Rt. Ant	10	Lt. IHD	300 + 120
8	80	Male	0.035	CCA	B-IIIA	2nd GW/0.025	Rt. Ant	5	Lt. IHD	300 + 60
9	93	Female	0.035	GB ca.	B-IV	2nd GW/0.025	Rt. Ant	50	Lt. IHD	300 + 270
10	89	Male	0.035	CCA	B-IV	1st GW/0.025	Rt. Ant	300 + 250	Lt. IHD	72 *
11	78	Female	0.035	CCA	B-IIIB	2nd GW/0.025	Rt. Ant	31	fail	300 + 300
12	50	Male	0.025	CCA	B-IIIA	2nd GW/0.035	Rt. Ant	30	fail	300 + 300
13	78	Male	0.025	CCA	B-IIIA	2nd GW/0.035	Rt. Ant	5	fail	300 + 300
14	78	Female	0.025	CCA	B-IIIA	2nd GW/0.035	Lt. IHD	5	fail	300 + 300
15	68	Female	0.025	CCA	B-IIIA	2nd GW/0.035	Lt. IHD	15	fail	300 + 300

* performed by 0.025 inch guidewire; B, Bismuth; Time (S) + alpha, cross over time; IHD, intrahepatic duct; CCA, cholangiocarcinoma; GB ca., gallbladder cancer; GW, guidewire.

## Data Availability

All relevant data contained within the article.
